# Diagnosis of Rejection by Analyzing Ventricular Late Potentials in Heart Transplant Patients

**DOI:** 10.5935/abc.20160011

**Published:** 2016-02

**Authors:** Vítor Nogueira Mendes, Telmo Santos Pereira, Vítor Azevedo Matos

**Affiliations:** 1 Centro de Cirurgia Cardiotorácica - Centro Hospitalar e Universitário de Coimbra, Coimbra - Portugal; 2 Departamento de Cardiopneumologia - Escola Superior de Tecnologia da Saúde de Coimbra, Coimbra - Portugal; 3 Serviço de Cardiologia - Centro Hospitalar e Universitário de Coimbra, Coimbra - Portugal

**Keywords:** Heart Transplantation, Graft Rejection, Endomyocardial Fibrosis, Electrocardiography

## Abstract

**Background:**

Heart transplant rejection originates slow and fragmented conduction. Signal-averaged ECG (SAECG) is a stratification method in the risk of rejection.

**Objective:**

To develop a risk score for rejection, using SAECG variables.

**Methods:**

We studied 28 transplant patients. First, we divided the sample into two groups based on the occurrence of acute rejection (5 with rejection and 23 without). In a second phase, we divided the sample considering the existence or not of rejection in at least one biopsy performed on the follow-up period (rejection pm1: 18 with rejection and 10 without).

**Results:**

On conventional ECG, the presence of fibrosis was the only criterion associated with acute rejection (OR = 19; 95% CI = 1.65-218.47; p = 0.02). Considering the rejection pm1, an association was found with the SAECG variables, mainly with RMS40 (OR = 0.97; 95% CI = 0.87-0.99; p = 0.03) and LAS40 (OR = 1.06; 95% IC = 1.01-1.11; p = 0.03). We formulated a risk score including those variables, and evaluated its discriminative performance in our sample. The presence of fibrosis with increasing of LAS40 and decreasing of RMS40 showed a good ability to distinguish between patients with and without rejection (AUC = 0.82; p < 0.01), assuming a cutoff point of sensitivity = 83.3% and specificity = 60%.

**Conclusion:**

The SAECG distinguished between patients with and without rejection. The usefulness of the proposed risk score must be demonstrated in larger follow-up studies.

## Introduction

Rejection is one major cause of death among heart transplant patients. According to the International Society for Heart and Lung Transplantation (ISHLT), 21% to 30% of heart transplant patients develop at least one episode of rejection within the first year from transplantation.^1^ In the Cardiothoracic Surgery Center of the Coimbra University-affiliated Hospital Center (CCT-CHUC), the prevalence of rejection is 10% in 8.5 years of clinical follow-up.^2^

Although studies have confirmed the efficacy of immunosuppressive therapy to prevent rejection, the diagnosis of that condition remains a challenge. Right ventricular (RV) endomyocardial biopsy is the standard method to diagnose rejection, but it is invasive and has inherent morbidities. To avoid the limitations of that methodology, there has been an effort to develop alternative methods, such as the use of biomarkers and echocardiographic assessment, to diagnose rejection.

Because rejection causes morphofunctional changes, mainly zones of myocardial fibrosis characterized by slow and fragmented electrical conduction,^3-6^ the presence of ventricular late potentials (VLP) on the signal-averaged ECG (SAECG) of such patients is considered a clinically relevant predictor.

A study performed with 20 transplanted individuals undergoing endomyocardial biopsy has shown a reduction in the RMS40 (terminal QRSf amplitude in the last 40 ms) and QRSf values of individuals with rejection as compared to individuals without rejection.^7^ Another study performing SAECG in 20 transplanted patients treated with cyclosporine has reported good reproducibility (r = 0.83) and good accuracy of that test in the late postoperative period to detect rejection.^8^ In a study investigating the relationship between VLP and acute rejection, Morocutti has reported sensitivity and specificity for the presence of VLP in cases of acute rejection of 69% and 71% respectively.^9^

Assessing VLP in transplanted patients is an important objective, motivating the performance of this study aimed mainly at assessing the use of that non-invasive technique to diagnose rejection in transplanted patients and at creating a risk score to predict episodes of rejection, based on clinical and electrophysiological parameters.

## Methods

### Sample

The size of the sample was based on convenience, considering the available population of transplanted patients and the technical conditions necessary to the intended clinical assessments within the time period established for data collection.

This study sample was formed by 28 heart transplant patients (23 men and 5 women) at the CCT-CHUC, with ages ranging from 19 to 76 years (mean age of 54 ± 12.22 years). This was an observational study of fixed cohort.

### Procedure

All patients underwent catheterization with RV endomyocardial biopsy, followed by ECG, SAECG and echocardiography. In the first phase, the following data were obtained: results of those exams, dosage and type of immunosuppressive therapy of each patient, and clinical analyses.

The right catheterization with RV endomyocardial biopsy was based on the modified Seldinger technique, via right (25 individuals) and left (3 individuals) femoral veins. All procedures were performed with a puncture needle (18G), a 7F introducer (9F in cases of important inguinal fibrosis), a 150-cm-long J-tip guidewire, 7F sheath, 7F pigtail catheter, and a 7F bioptome. In each procedure, two fragments of the right interventricular septum were collected for microscopic examination at the service of Anatomic Pathology of the CHUC, and the criteria of the ISHLT 2004 formulation were used for the diagnosis of rejection.^10^

In the post-catheterization resting period, 12-lead conventional ECG and SAECG were performed in a calm and noise-free environment, using NORAV-ECG Monitoring Version 5.0.2 (Norav Medical Ltd).

The SAECG tracing was obtained using the Frank lead system, with the orthogonal X, Y, Z lead configuration.

The tracing was obtained by using the arithmetic mean of the sum of 200 identical QRS complexes (95% of correspondence) collected in 4 minutes, which, after a filtering process, increased the signal-noise ratio of the complexes collected, evidencing low-amplitude and long-duration signals, known as VLP.

After the post-catheterization resting period, patients underwent two-dimensional echocardiography.

In the second phase, the following data were obtained: demographics; reason for heart transplantation; NYHA functional class relative to the severity of each patient's symptoms; antecedents and cardiovascular risk factors; comorbidities; left ventricular function by using echocardiography, radionuclide ventriculography and angiography prior to transplantation; respiratory capacity prior to transplantation; intracavitary pressures and coronary angiography prior to transplantation.

In addition, demographics of heart donors, their cause of death and histocompatibility with recipients were obtained, as well as data regarding surgical times.

The diagnosis of rejection on endomyocardial biopsy, on the day of the ECG recording, indicated the criterion to divide the sample. Thus, on the first phase of our investigation and based on the result of the endomyocardial biopsy on the day of the ECG recording, the sample was divided into two groups: one group whose biopsy showed no rejection; and another group whose biopsy showed rejection.

On the second phase, the group division remained, but considering the diagnosis of rejection in at least one biopsy performed until the day of ECG recording (rejection pm1).

The sampling technique was based on the recognition of certain characteristics of the patients, such as performance of endomyocardial biopsy, to ensure the best representativeness possible, being then a non-probabilistic sampling, a convenience sampling.

Because the selection criteria can limit the probabilistic character of the sample, inclusion and exclusion criteria were established. All patients aged at least 18 years, heart transplanted and undergoing endomyocardial biopsy within the last 24 hours were included.

All pacemaker users, as well as those not meeting the inclusion criteria, were excluded.

Regarding the ethical questions of this investigation, it is worth noting that the data collected were exclusively aimed at carrying out this scientific study, with protection of the anonymity of all individuals. There was no commercial interest.

### Statistical analysis

After data collection, statistical analysis was performed with the Statistical Package for the Social Sciences (SPSS) program, version 13.

At the initial phase, a simple descriptive analysis was performed, with the calculation of mean ± standard deviation and relative and absolute frequencies to characterize the variables of the sample.

To assess the normality of the distribution of the continuous variables, Shapiro-Wilks test was used. In the presence of a normal distribution, parametric statistical tests were performed, and in its absence, non-parametric statistical tests were performed. To compare continuous variables between the two groups, Student *t* test was used for independent samples or Mann-Whitney U test.

To compare categorical variables, the chi-square test was used, the Fisher Exact test being used when the number of cases in any cell of the contingency table was lower than 5.

Regarding hypothesis testing, Spearman rank correlation and Cohen's kappa agreement were used.

To identify predictors of rejection, univariate analysis was used to enable the elaboration of a risk score.

The receiver operating characteristic (ROC) curve was used to assess the performance of each predictor and of the formulated score. The sensitivity and specificity values were analyzed for each cutoff point.

The statistical tests were interpreted based on the significance level of α = 0.05 with 95% confidence interval (CI); however, for the elaboration of the risk score, the significance level of α = 0.1 with 95% CI was adopted.

## Results

We studied 28 heart transplant patients, 5 of whom had biopsy findings of acute rejection.

Clinical, demographic, echocardiographic, electrocardiographic and hemodynamic variables were compared between the groups with and without acute rejection.

Acute rejection only evidenced association with the presence of signs of fibrosis on ECG. By using logistic regression, we observed that the presence of fibrosis on ECG increases 19 times the risk of acute rejection (OR = 19; 95% CI = 1.65-218.47; p = 0.02).

**Figure 1:**
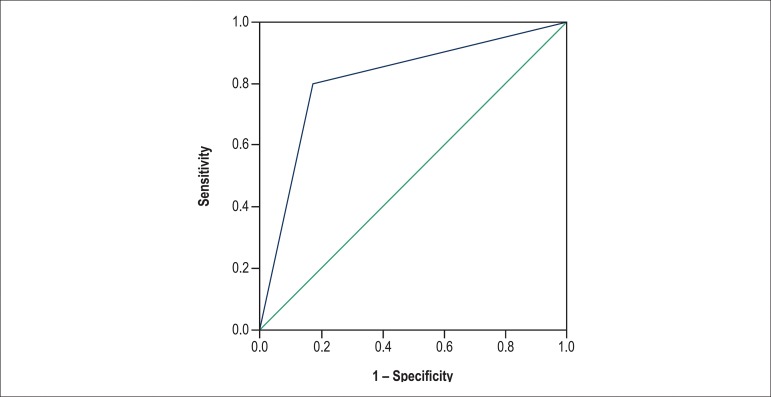
ROC Curve for the association between fibrosis and acute rejection.

By using a ROC curve, the strength of the association of fibrosis and acute rejection was assessed. A cutoff point was identified with sensitivity of 80%, specificity of 82.6% and AUC of 0.81, indicating a good ability to discriminate between transplanted patients with and without rejection (p = 0.03) (Figure 1).

Because fibrosis was the only variable to show association with the diagnosis of acute rejection, the elaboration of a risk score was impossible. Thus, the variables in this study were compared considering a positive diagnosis of rejection in at least one endomyocardial biopsy performed (rejection pm1).

The prevalence of rejection pm1 was 64.2%, and significant differences were found between patients with and without rejection pm1 regarding some variables.

All variables with a significance level below 10% were selected, excluding those with the number 1 in the 95% CI.

Considering the continuous variables, only RMS40 and LAS40 (duration of the low amplitude electric potential component < 40 µV) showed an association with rejection pm1.

For each unit reduction in RMS40, there is a 7% increase in the risk of rejection (OR = 0.93; 95% CI = 0.87-0.99; p = 0.03).

In addition, the increase in LAS40 showed a 1.06-fold increase in the risk of rejection (OR = 1.06; 95% CI = 1.01-1.11; p = 0.03).

Considering the categorical variables, more precisely the general criteria of the VLP diagnosis, we observed that only the criteria formulated by the American College of Cardiology^11^ (ACC) evidenced association with the diagnosis of rejection pm1. In the presence of VLP, using the ACC criteria, the risk of rejection increases 7.5 times (OR = 7.5; 95% CI = 1.27-44.09; p = 0.03).

Regarding individual criteria, those evidencing the strongest associations with rejection pm1 were RMS40 (according to Narayanaswamy^11^ and Brembilla-Perrot et al)^12^ and LAS40 (according to Gatzoulis et al),^13^ with a 7.5-fold increase in the risk of rejection when RMS40 is equal to or lower than 20 µV (OR = 7.5; 95% CI = 1.28-44.09; p = 0.03), and a 14.14-fold increase in that risk when LAS40 is equal to or greater than 50ms (OR = 14.14; 95% CI = 1.46-137.30; p = 0.02).

### Elaboration of the risk score

Considering the logistic regression data, and that more than one variable showed to be associated with the diagnosis of rejection, we decided to elaborate a risk score for rejection pm1.

According to the ACC criteria, when RMS40 ≤ 20 µV and LAS40 ≥ 38 ms, there is a positive diagnosis for the presence of VLP.

Thus, we initially elaborated a score combining the ACC general criteria for the presence of VLP and the increasing values of LAS40. The point attribution considered the approximate value of OR for each variable (Table 1).

**Table 1:** Point attribution of the criteria included in SCORE1

Criteria		Points
ACC	Yes	8
	No	0
LAS40	≥ 50	14
	[45-50[	8
	[40-45[	8
	[38-40[	8
	[30-38[	5

ACC: American College of Cardiology; LAS40: Terminal duration of QRSf < 40 µV.

Thus, according to ACC criteria, because OR was 7.5, we attributed 8 points to the presence of VLP. In the absence of VLP, we attributed 0 point.

Regarding LAS40, using OR values, point attribution obeyed the same principle.

Based on that, each patient was attributed points, yielding a risk score: SCORE1 = ACC + LAS40.

**Figure 2:**
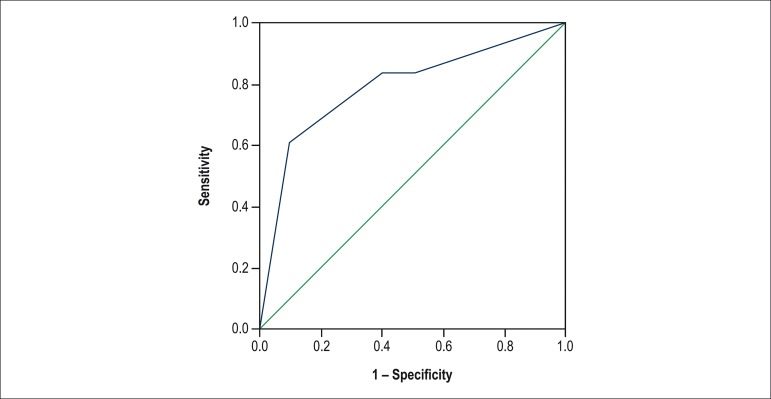
ROC Curve for the association of SCORE1 with rejection pm1.

To assess the predictive ability of SCORE1, a ROC curve was built, showing, for a cutoff point with sensitivity of 83.3% and specificity of 60%, good ability to discriminate between patients with and without rejection pm1 (AUC = 0.79; p = 0.01) (Figure 2).

Although that result was satisfactory, we considered elaborating a new score by adding RMS40 values to SCORE1. However, we noticed that the values added were already implicit in the points attributed according to the ACC general criteria.

Considering that the presence or absence of the ACC criteria could only allow the attribution of two point values, we decided to elaborate SCORE 2 resorting to RMS40 values. Thus, SCORE2 = RMS40 + LAS40.

Although RMS40 ≤ 20 µV was the only signal-amplitude-linked variable to show association with the diagnosis of rejection pm1, the values of RMS40 ≤ 17.5 (OR = 3.9; 95% CI = 0.76-19.95; p = 0.10) and RMS40 ≤ 15 (OR = 4.67; 95% CI = 0.88-24.80; p = 0.07) were added to SCORE2, because they tended to be significant.

Point attribution only considered the OR values for RMS40 ≤ 20 µV. For the two other conditions, due to RMS40 decrease, one more point was attributed, as shown in Table 2.

**Table 2:** Point attribution of the RMS40 criteria included in SCORE2

Criteria		Points
RMS40	> 20	0
	[17.5-20[	8
	[15-17.5[	9
	< 15	10

RMS40: Terminal QRSf amplitude in the last 40 ms.

**Figure 3:**
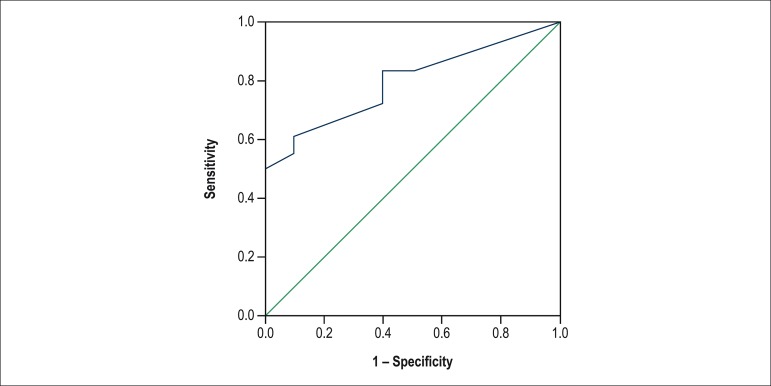
ROC curve for the association of SCORE2 with rejection pm1.

After calculating the SCORE2 for each patient, the predictive ability of that score was assessed by use of a ROC curve (Figure 3).

SCORE 2 showed a good ability to discriminate between patients with and without rejection pm1, using a cutoff point with sensitivity of 83.3% and specificity of 60% (AUC = 0.79; p = 0.01).

After using SCORE 2, a new score was elaborated by adding to it the variables that, according to our analysis, could improve the discriminatory character of the ROC curve.

Logistic regression showed no association of the variable 'fibrosis' with the diagnosis of rejection pm1.

However, the Fisher test (χ2 = 6.22; p = 0.03) complemented by the Spearman correlation (Rho = 0.47; p = 0.01) showed a moderate and positive relationship between fibrosis and rejection pm1, that variable being thus added to the new score formulated.

To patients with signs of fibrosis on conventional ECG, 1 point was attributed, and to those without them on conventional ECG, none.

Thus, SCORE3 = SCORE2 + Fibrosis.

**Figure 4:**
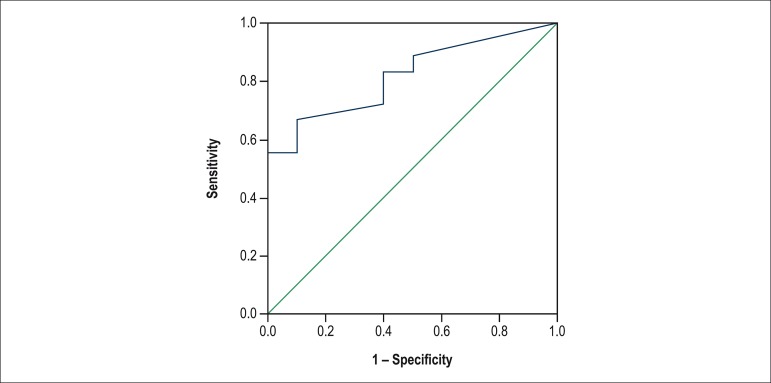
ROC curve for the association of SCORE3 with rejection pm1.

To assess the discriminatory ability between patients with and without rejection pm1, a ROC curve was built (Figure 4). Assuming a cutoff point with sensitivity of 83.3% and specificity of 60% (AUC = 0.82; p < 0.01), a good ability to distinguish between the two groups studied was demonstrated.

The ROC curve for SCORE3 showed the greatest discriminatory power to diagnose rejection pm1.

Based on the various cutoff points, different levels of probability of the occurrence of rejection pm1 could be assessed (Table 3).

**Table 3:** Relationship between point attribution and the probability of the diagnosis of rejection pm1 according to SCORE3

Points	Sensitivity	Specificity	Probability of the diagnosis of rejection pm1
≥ 23	55.6%	100%	Very strong probability
[18-23[	66.7%	90%	Strong probability
[11-18[	83.3%	60%	Moderate probability
< 11	83.3%	50%	Reduced probability

The analysis of Table 3 shows that all patients with a SCORE3 of at least 23 points have a strong probability of rejection. However, for a score lower than 11, that is a less likely diagnosis.

## Discussion

This study aimed at assessing the usefulness of SAECG as a method to diagnose heart graft rejection by elaborating a risk score.

Similarly to that existing in some studies for the diagnosis of channelopathies, this study attempted to elaborate a diagnostic test based on a score of the probability of rejection.

Considering the result of the endomyocardial biopsy on the day of ECG recording, the prevalence of acute rejection was 17.9%.

Initially, the groups significantly differed regarding the diverse biochemical and electrophysiological variables. However, and probably because of the low statistical power of this study, due to its reduced sample size, only the variable 'fibrosis' associated, on logistic regression, with the diagnosis of acute rejection, revealing a 19-fold increase in the risk when present on the ECG recordings (OR = 19; 95% CI = 1.65-218.47; p = 0.02). This is in accordance with other studies that indicate that the appearance of myocardial fibrosis strongly relates to acute rejection.^4^ The process of acute rejection has been reported to occur as soon as the blood flow is reestablished in the coronary arteries and even in the transplanted heart itself.^14^ According to Chassot et al,^4^ the reaction caused by the attraction of alloreactive T lymphocytes against graft antigens, associated with ischemia-reperfusion lesions instituted with blood flow reestablishment to the coronary arteries, will determine cell changes that culminate in zones of fibrosis. With widened periods of ischemia, ATP and glycogen are depleted due to lack of oxygen, leading to mitochondrial edema. Rupture of the mitochondrial crests occur, and the Krebs cycle is interrupted. Catalyzation of energy-rich compounds, such as fatty acids, begins, increasing cell osmolality. Ca^2+^, which had left the sarcoplasmic reticulum to intervene in systole, is no longer reabsorbed due to lack of ATP, remaining in the cell cytoplasm. With reperfusion, in addition to the sudden O_2_ offer that originates free radicals responsible for acidosis of the intracellular medium, there is edema of the myocyte, which, due to the high osmotic gradient, leads to entrance of H_2_O and ions (Na^2+^, Ca^2+^).^4^

The excess of free radicals, associated with the accumulation of Ca^2+^ and H^+^ in the cytoplasm of the myocyte, leads to changes in cell organization and functionality. In addition, degradation of connexins 43 has been reported, hindering the conduction of the electrical stimulus between myocytes.^5^ Thus, the cardiac tissue transforms into connective tissue, leading to the appearance of zones of fibrosis of slow conduction that manifest as VLP on the SAECG. The increase in the zones of fibrosis hinders myocardial contractility, and graft loss becomes inevitable.

In this study, we attempted to assess the relationship between VLP and the diagnosis of acute rejection. The lack of statistical significance, possibly due to the reduced sample size, made the demonstration of that statement impossible. However, a significant reduction could be observed in the absolute values of RMS40 in individuals with rejection as compared to those without rejection.

Those results are in accordance with those of the study by Graceffo and O'Rourke in 1996, which, in a population of 20 heart transplant patients, also reported a decrease in RMS40 in those with rejection.^7^

In addition, an increase in QRSf and LAS40, as well as in the number of QRSf notching, was observed in the group of patients with rejection, emphasizing the positive diagnosis of VLP, according to the ACC criteria.

Because of the low statistical value of most variables, the elaboration of a test to diagnose acute rejection was difficult.

Based on the assumption that the zones of fibrosis remain, even with the increase in immunosuppression, after a positive diagnosis of rejection on an endomyocardial biopsy, in a second phase of the investigation, the sample was divided into two groups considering the presence or absence of rejection on at least one biopsy, from transplantation until the day of ECG recording (rejection pm1). The proportion of patients with at least one diagnosis of rejection until the date of assessment was 64.2%. Several variables differed significantly between the groups with and without rejection pm1. To assess the strength of the relationship between the variables considered and the probability of rejection pm1, simple logistic regression was performed. We identified that the ACC criteria had a moderate ability to discriminate between transplanted individuals with and without rejection pm1 (AUC = 0.72; p = 0.06). In addition, similarly to the decrease in RMS40 (OR = 7.5; 95% CI = 0.87-0.99; p = 0.03), the increase in LAS40 increased the risk of rejection pm1 (OR = 1.06; 95% CI = 1.0-1.11; p = 0.03).

Based on that information and on the normal VLP values reported in the literature, we could identify, by use of a ROC curve, several cutoff points to enable the elaboration of a probability score of rejection pm1. Point attribution enabled the stratification of the probability of rejection, as an indicator of the risk for the occurrence of such an important clinical event in that population. We believe that this clinical decision tool enables the discrimination of patients who will require endomyocardial biopsy to confirm the process of rejection, when the score indicates a significant probability of rejection. On the other hand, a low probability of rejection according to the score will spare patients from that invasive procedure, with all inherent benefits regarding possible complications and quality of life in general. This optimizes the decision for those patients and strongly reduces their load of percutaneous procedures during clinical follow-up, with significant advantages not only regarding a reduction in potential complications and the suffering associated, but also in terms of direct and indirect costs.

This study is the first attempt to elaborate an instrument of clinical decision making to properly and accurately screen rejection in heart transplant patients. However, this study has important limitations, which, despite their relevance, make the results suitable for the preliminary development of the score proposed.

The first major limitation refers to the lack of uniformity of the normality criteria for the diagnosis of VLP based on SAECG. Although the ACC criteria have achieved more consensus within the scientific community, their validity is conditioned by the duration of the QRS complex (< 120 ms). Thus, considering the various studies revealing the presence of right bundle branch block in 80-90% of heart transplant patients^14^, other criteria were incorporated in this investigation allowing the validation of the SAECG recordings in patients with bundle branch block.

The reduced sample size, associated with the reduced number of acute rejection processes, proved to be an important limitation, determining the reduced global statistical power of this study. Thus, the results should be read with due caution, the study replication being fundamental, as well as the verification of the accuracy and reliability of the score proposed in a larger study. The lack of technology for the assessment of atrial late potentials is also a limitation, because the incorporation of that component in the analysis could add discriminative ability to the score, an aspect that remains to be demonstrated. On the other hand, the incorporation of other laboratory variables can contribute to increase the robustness of the score proposed, an aspect assumed as a challenge to be considered, providing unequivocal clinical benefit for transplant patients on the existence of a non-invasive instrument to identify those effectively in need for endomyocardial biopsy, thus avoiding unnecessary percutaneous procedures and all their inherent complications and costs.

## Conclusion

The SAECG is an effective tool to sort patients out according to the presence or absence of rejection.

We observed that the presence of signs of myocardial fibrosis on ECG is strongly associated with an increase in the risk of acute rejection, and that a reduction in RMS40 on SAECG tends to relate to that diagnosis.

In addition, the ACC criteria have a moderate ability to discriminate between transplanted individuals with and without rejection pm1, and that, similarly to the reduction in RMS40, the increase in LAS40 increases the risk of rejection pm1.

Based on that information, we elaborated a probability score of rejection pm1, which allows the stratification of the probability of rejection.

Although the usefulness of SAECG is camouflaged against acute rejection, probably because of its low statistical power, SAECG has a great value to preview rejection pm1. The potential usefulness of the score should be demonstrated in follow-up studies with a larger sample size.
